# Investigation of AlGaN/GaN high electron mobility transistor structures on 200-mm silicon (111) substrates employing different buffer layer configurations

**DOI:** 10.1038/srep37588

**Published:** 2016-11-21

**Authors:** H.-P. Lee, J. Perozek, L. D. Rosario, C. Bayram

**Affiliations:** 1 Department of Electrical and Computer Engineering, University of Illinois at Urbana-Champaign, Urbana, Illinois 61801, USA; 2 Micro and Nanotechnology Laboratory, University of Illinois at Urbana-Champaign, Urbana, Illinois 61801, USA

## Abstract

AlGaN/GaN high electron mobility transistor (HEMT) structures are grown on 200-mm diameter Si(111) substrates by using three different buffer layer configurations: (a) Thick-GaN/3 × {Al_x_Ga_1−x_N}/AlN, (b) Thin-GaN/3 × {Al_x_Ga_1−x_N}/AlN, and (c) Thin-GaN/AlN, so as to have crack-free and low-bow (<50 μm) wafer. Scanning electron microscopy, energy-dispersive X-ray spectroscopy, high resolution-cross section transmission electron microscopy, optical microscopy, atomic-force microscopy, cathodoluminescence, Raman spectroscopy, X-ray diffraction (ω/2*θ* scan and symmetric/asymmetric ω scan (rocking curve scan), reciprocal space mapping) and Hall effect measurements are employed to study the structural, optical, and electrical properties of these AlGaN/GaN HEMT structures. The effects of buffer layer stacks (i.e. thickness and content) on defectivity, stress, and two-dimensional electron gas (2DEG) mobility and 2DEG concentration are reported. It is shown that 2DEG characteristics are heavily affected by the employed buffer layers between AlGaN/GaN HEMT structures and Si(111) substrates. Particularly, we report that in-plane stress in the GaN layer affects the 2DEG mobility and 2DEG carrier concentration significantly. Buffer layer engineering is shown to be essential for achieving high 2DEG mobility (>1800 cm^2^/V∙s) and 2DEG carrier concentration (>1.0 × 10^13^ cm^−2^) on Si(111) substrates.

AlGaN/GaN high electron mobility transistors (HEMTs) are being investigated for high power high frequency applications as III-nitride (i.e. GaN) materials have high thermal and chemical stability, high breakdown field (>3 MV/cm, 10 times of that of silicon), and high electron saturation velocity (>2.5 × 10^7^ cm/s, 2.5 times of that of silicon)^1^,^2^,^3^. AlGaN/GaN HEMTs are traditionally grown on sapphire (Al_2_O_3_) or silicon carbide (6H-SiC) substrates that have ~16% and ~3% lattice-mismatch with GaN, respectively^4^. Recently, high cost and limited diameter-scalability of these substrates fueled the research for the GaN-on-silicon (111) approach^5^. However, the lattice mismatch of ~17% combined with the thermal-expansion-coefficient mismatch of ~54% between GaN and Si(111) necessitate employment of novel (Al)GaN buffer layers to minimize mismatch-effects (i.e. defectivity and wafer-bow)^6^. It is reported that several micrometer thick buffer layers and various Al-content Al_x_Ga_1−x_N layers are needed to mitigate such detrimental effects of lattice and thermal-mismatches^7^. Nonetheless, performance of AlGaN/GaN HEMTs is governed by the two-dimensional electron gas (2DEG) properties, which forms at the AlGaN-GaN hetero-interface and without the need of any doping – thanks to the high conduction band offset and polarization fields between AlGaN and GaN^8^. Particularly, a high 2DEG density reduces the source/drain contact resistance^9^ and increases the power output of AlGaN/GaN HEMTs^3^ whereas high 2DEG mobility increases the frequency performance of AlGaN/GaN HEMTs^10^. It is therefore imperative to study the characteristics of the 2DEG and investigate how 2DEG characteristics change under various buffer layer configurations. To do so, the same AlGaN/GaN HEMT structures need to be grown on Si(111) but with various buffer layers^7^.

Another important milestone in GaN-on-Si(111) technology is Si(111) wafer-scaling. Despite the early works on 100-mm substrates^11^, it is critical to scale these efforts to 200-mm substrates. This, however, is bottlenecked primarily by the large GaN-Si thermal mismatch that introduces high stress leading to significant wafer-bow^12^ or worse, wafer cracking^13^.

In this work, we grew the same AlGaN/GaN HEMT structures on 200-mm Si(111) substrates using three different buffer layers configurations {such that all wafers are crack-free and have a small bow (<50 μm)} and report the effects of buffer layers on the AlGaN/GaN HEMT structures. To quantify the stress and defectivity, we investigate these stacks using structural, optical and electrical characterization techniques including scanning electron microscopy (SEM), energy-dispersive X-ray spectroscopy (EDS), high resolution cross-sectional transmission electron microscopy (HR-XTEM), optical microscopy, atomic-force microscopy (AFM), cathodoluminescence (CL), Raman spectroscopy, X-ray diffraction (XRD) {ω/2*θ* scan, symmetric/asymmetric ω scan (rocking curve scan), and reciprocal space mapping (RSM)} and Hall effect measurements. Then we correlate the electrical properties of AlGaN/GaN HEMT structures with the embodied buffer layer properties and report the effects of buffer layer stress and defectivity on the 2DEG mobility and 2DEG concentration.

## Results

### Investigating the AlGaN/GaN HEMT structures and the underlying buffer layer configurations

**Figure 1:**
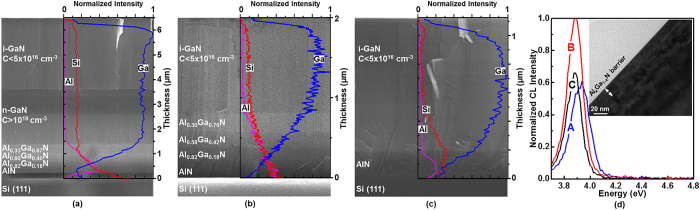
SEM cross section, EDS, HR-XTEM, and CL investigations of the AlGaN/GaN HEMT structures grown on Si(111) substrate with three different buffer configurations are shown. (**a**) Thick-GaN/3 × {Al_x_Ga_1−x_N}/AlN (Sample A), (**b**) Thin-GaN/3 × {Al_x_Ga_1−x_N}/AlN (Sample B), and (**c**) Thin-GaN/AlN (Sample C). Atop of all samples (A, B, C), the same AlGaN/GaN HEMT structure, composed of 2-nm-thick i-GaN/17-nm-thick Al_x_Ga_1−x_N/1-nm-thick AlN, is deposited as shown in (**d**). Based on the CL spectrum peak position of the Al_x_Ga_1−x_N barrier layer, Al composition can be estimated as 0.25, 0.23, and 0.22 for sample A, B, and C, respectively.

Figure 1 shows the AlGaN/GaN HEMT structures grown on Si(111) substrate with three different buffer configurations: (a) Thick-GaN/3 × {Al_x_Ga_1−x_N}/AlN (Sample A), (b) Thin-GaN/3 × {Al_x_Ga_1−x_N}/AlN (Sample B), and (c) Thin-GaN/AlN (Sample C). Hitachi S-4700/S-4800 high resolution SEM is used to measure layer thicknesses. All three configurations employ an AlN buffer layer (240-nm-thick, 175-nm-thick, and 130-nm-thick in samples A, B, and C, respectively) on top of the Si(111) substrate to prevent Ga-etch back during (Al)GaN growth^14^. On top of the AlN buffer layers, samples A and B have three step-graded Al_x_Ga_1−x_N buffer layers (400-nm-thick Al_0.33_Ga_0.67_N/290-nm-thick Al_0.60_Ga_0.40_N/200-nm-thick Al_0.82_Ga_0.18_N in sample A and 240-nm-thick Al_0.30_Ga_0.70_N/210-nm-thick Al_0.58_Ga_0.42_N/190-nm-thick Al_0.82_Ga_0.18_N in sample B) whereas sample C has no AlGaN buffer layer. Then, 2280-nm-thick n-doped (n-) followed by 2880-nm-thick intrinsic (i-) GaN layers were grown for sample A whereas only 1200- and 950-nm-thick intrinsic GaN layers were deposited on samples B and C, respectively. Atop of all samples, the same AlGaN/GaN HEMT structure, composed of 2-nm-thick i-GaN/17-nm-thick Al_x_Ga_1−x_N/1-nm-thick AlN, was deposited. Figure 1d shows HR-XTEM and CL of the AlGaN/GaN HEMT structure. Intrinsic GaN cap protects the Al_x_Ga_1−x_N barrier layer surface and minimizes the electrical contact resistance for Hall measurements^15^. To minimize layer relaxation through defect generation at the Al_x_Ga_1−x_N-GaN hetero-interface, only a 17-nm-thick Al_x_Ga_1−x_N barrier layer (measured by HR-XTEM) is grown^16^. We also employed CL spectroscopy (JEOL 7000 F analytical Schottky field emission SEM equipped with Gatan MonoCL3 CL Spectrometer) to probe the bandgap energy of the Al_x_Ga_1−x_N barrier layer for estimating the Al composition. Cathodoluminescence measurement was conducted with 5x magnification, 1.5 kV electron acceleration voltage (which yields a 29-nm penetration depth based on Kanaya-Okayama formula^17^), and 2 nA current. The measured spectrum was fitted using Gaussian distribution and the peak positions were obtained as 3.924 eV, 3.882 eV, and 3.874 eV for sample A, B, and C, respectively. Based on the peak position, we calculated the Al composition (x) of the Al_x_Ga_1−x_N barrier layer to be 0.25, 0.23, and 0.22 for sample A, B, and C, respectively^18^. The AlN spacer layer is inserted between the Al_x_Ga_1−x_N barrier and GaN to increase the conduction band offset and the 2DEG confinement in order to increase 2DEG concentration and mobility^19^,^20^. Overall, samples A, B, C have the total epilayer thickness of 6.3, 2.0, and 1.2 μm, respectively (Fig. 1).

The critical elemental compositions (i.e. Al, Ga, Si) across these samples (i.e. A, B, C) are investigated by Oxford Instrument ISIS EDS X-ray microanalysis system with a spatial resolution of ~1 μm and plotted in Fig. 1. In samples A and B, from GaN layer towards the Si(111) substrate, the Ga signal decreases as the Al signal increases, as we have step-graded Al_x_Ga_1−x_N layers. In sample C a rather rounded Al signal peak is observed as it has no step-graded Al_x_Ga_1−x_N layers. The Si signal increases in all samples towards the substrate showing the Si diffusion.

### Surface investigation of the AlGaN/GaN HEMT structures via optical microscope, atomic force microscopy, and cathodoluminescence

**Figure 2:**
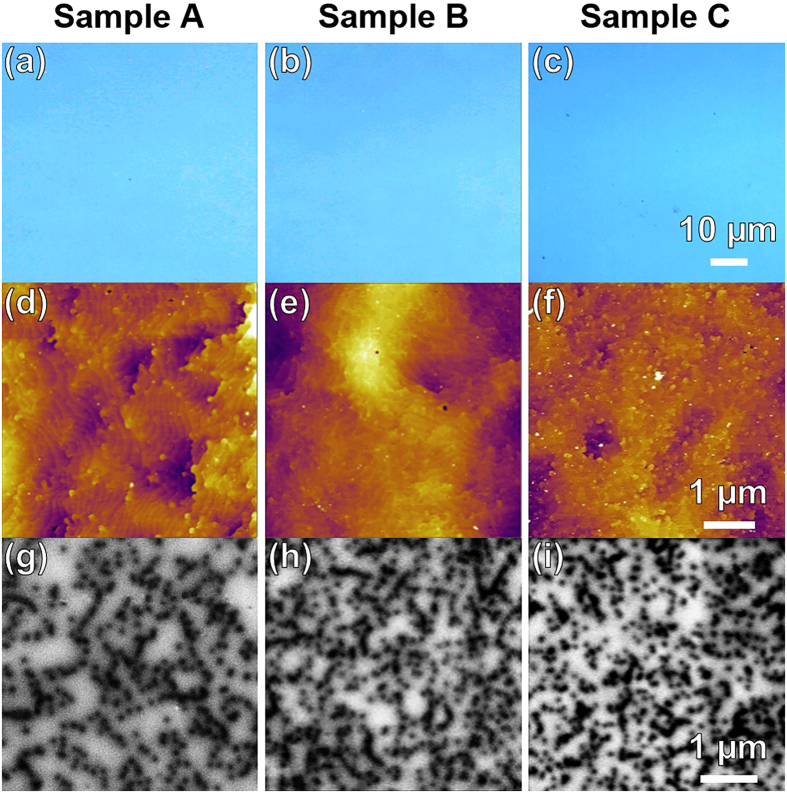
Optical microscopy, AFM and CL images of samples A, B and C are shown. Optical microscopy (**a**,**b**,**c**) reveals no surface cracks. AFM (**d**,**e**,**f**) reveals the average root-mean-square (RMS) roughness of 5.5 ± 2.8, 5.2 ± 1.4, and 5.1 ± 1.3 Å for sample A, B, and C, respectively, suggesting a similar surface profile. Cathodoluminescence (**g**,**h**,**i**) reveals defectivity of sample A, B, and C as 1.0 ± 0.3 × 10^9^, 2.0 ± 0.3 × 10^9^, 1.8 ± 0.3 × 10^9^ cm^−2^, respectively. AFM and CL studies agree that sample A (the thickest sample) has the lowest threading dislocation defect density whereas sample C (the thinnest sample) has the highest one (Table 1).

Figure 2 shows the surface studies of all samples (A, B, C) by optical microscopy (Fig. 2a,b,c), atomic force microscopy (Fig. 2d,e,f) and CL (Fig. 2g,h,i). No surface cracks are observed in any samples, indicating high film quality^13^. Atomic force microscopy (Asylum Cypher S AFM with tapping mode) is used to study the surface roughness and contour of the AlGaN/GaN HEMT structures. Figure 2d,e,f show 5 μm × 5 μm AFM scans. It is important to note that the average root-mean-square (RMS) roughnesses are similar across samples (5.5 ± 2.8, 5.2 ± 1.4, and 5.1 ± 1.3 Å, for A, B, and C, respectively), wherein sample A has the largest surface roughness. In order to quantify surface-terminated defects (i.e. dislocations with threading component), we counted the “dark spots” on the AFM images (Fig. 2d,e,f)^21^ which averaged as 1.8 ± 0.3 × 10^9^, 2.2 ± 0.8 × 10^9^, and 2.6 ± 0.8 × 10^9^ cm^−2^ (Table 1) for samples A, B, and C, respectively, showing thickest sample (A) having the lowest density of threading type dislocations, in agreement with other works^22^,^23^. Based on their different sizes these dark spots can be classified as pure-edge type (small), pure-screw type (large) and mixed-type (middle) threading dislocations.

Cathodoluminescence is also a useful tool in highlighting the surface defects as defects are centers of non-radiative recombination and appear dark in a panchromatic view^24^. We again employed the same CL spectroscopy system to study these samples. A panchromatic CL image using a 2.0-kV electron acceleration voltage (that gives a penetration depth of 47 nm^17^) reveals the defect distribution as shown in Fig. 2g,h,i. The average CL defectivity for samples A, B, and C are measured as 1.0 ± 0.3 × 10^9^, 2.0 ± 0.3 × 10^9^, and 1.8 ± 0.3 × 10^9^ cm^−2^ (Table 1), respectively. This suggests, similar to our AFM results, that the thickest sample A has the least defectivity. With respect to AFM analysis, CL underestimates the defectivity because defects have capture radius, suggesting that multiple defects are located within one CL dark capture radius.

### Layer stress analysis through Raman measurements, X-ray diffraction and reciprocal space mapping

**Figure 3:**
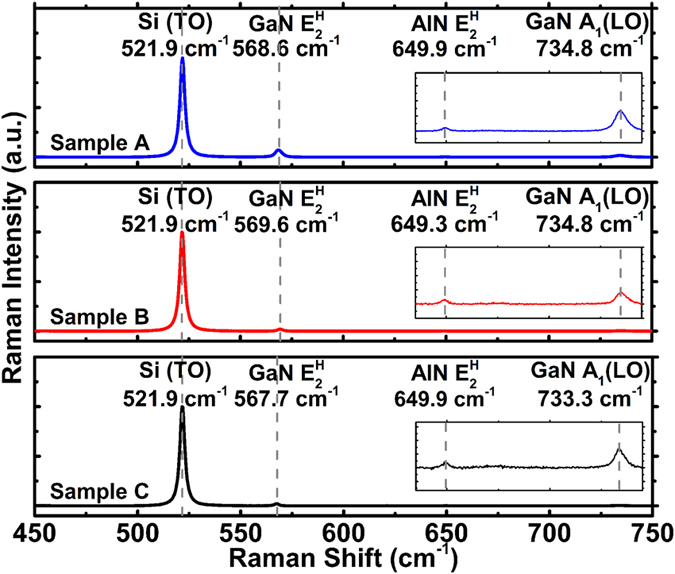
Raman spectroscopy of all samples A, B, and C are shown.

We studied the GaN stress via Horiba Raman Confocal Imaging Microscope using 633-nm laser line (with a grating of 1800 lines/mm, yielding a resolution of 1.19 cm^−1^). A 2″ Si(111) wafer was first utilized for spectrum verification and a 521.9 cm^−1^ peak was obtained, as shown in Figure S1. Under 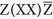 configuration, 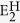 and A_1_(LO) Raman peaks are measured, and the shifts in these Raman peaks from the stress-free values of 567.5 cm^−1^ (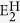) at 300 K are recorded. Based on the shift direction and the shift amount (∆ω) of 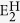, we determined the stress type as compressive and calculated the stress amount (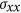) through^11^


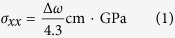


and tabulated them in Table 1. The Raman spectra of sample A, B, and C are also plotted in Fig. 3. Our Raman spectroscopy investigation suggests that sample B is under the largest compressive stress (0.488 GPa) whereas sample C is under the smallest compressive stress (0.047 GPa) and sample A has the medium value (0.256 GPa). These results are similar to other Raman works on AlGaN/GaN HEMT structures on Si(111)^11^.

**Figure 4:**
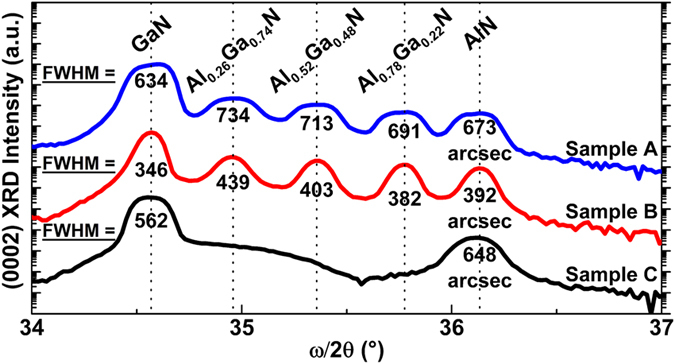
Symmetric XRD ω/2*θ* scans of samples A, B, and C are used to determine the Al-content and crystalline quality. Results show sample B having the narrowest FWHM values – suggesting the best crystallography quality amongst all samples.

We further quantify the (Al)GaN layers’ composition, strain, and defect density via XRD^25^,^26^. Here we employ PANalytical/Philips X’pert MRD system with 0.154-nm-wavelength radiation to study all samples. Omega/2Theta (ω/2*θ*) scan is used to probe the symmetrical lattice plane (0002) to determine the Al compositions of the Al_x_Ga_1−x_N buffer layers according to:


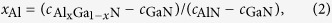


where *c* represents the lattice constant along the *c* axis calculated via Vegard’s law^27^. Figure 4 shows the ω/2*θ* scans of samples A, B, and C. The narrowest FWHM of sample B indicates the highest crystalline order amongst the samples, whereas sample A has the lowest crystalline order indicated by its widest FWHM. However, one should be aware that the Al composition results obtained from XRD ω/2*θ* scan can be inaccurate since both the epilayer strain and alloy composition will significantly affect the peak position of the ω/2*θ* scan; to estimate the Al composition more precisely, XRD reciprocal space mapping is necessary.

In addition, we conducted (0002) (symmetric) and 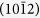 (asymmetric) *ω* scans to estimate the densities of screw-type (Burgers vector length: 0.5185 nm (*b*_screw_)) and edge-type (Burgers vector length: 0.3189 nm (*b*_edge_)) threading dislocations, respectively (Figure S2)^25^,^28^,^29^. Based on the FWHM of (0002) (symmetric) and 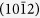 (asymmetric) *ω* scans (i.e. 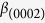 and 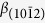), screw- and edge-type threading dislocation densities (*D*_screw_ and *D*_edge_) are estimated via^25^,^28^,^29^:


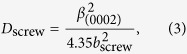



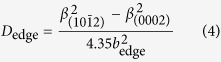


and tabulated in Table 1. Our XRD analysis suggests that sample A and C have similar amount of pure-edge- and screw-type threading dislocation densities.

**Figure 5:**
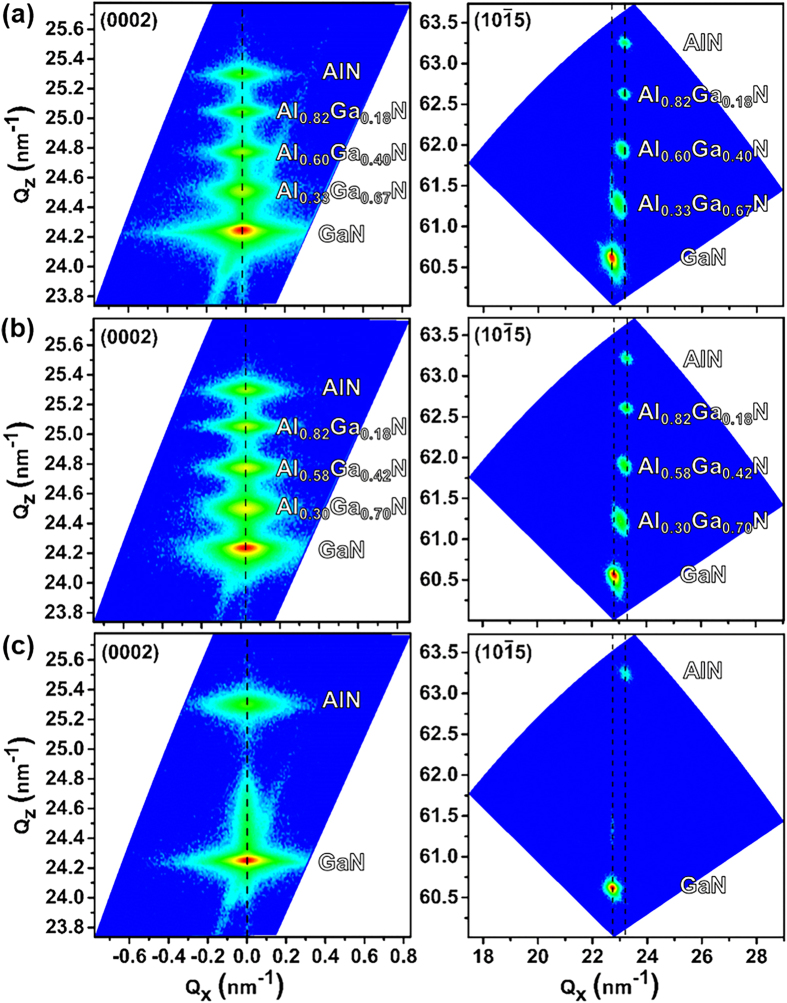
XRD reciprocal space mapping results reveal that the GaN layers of all samples (A, B, C) are under tensile strain along the *c*-plane and are under compressive strain normal to the *c*-plane. Amongst, sample B is observed to have the lowest GaN in-plane stress (Table 1).

X-ray diffraction reciprocal space mappings of (0002) and 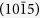 planes (Fig. 5) are performed to quantify the (Al)GaN layers’ strain and stress; in addition, the Al composition can be more precisely quantified compared to the ω/2*θ* scan. Lattice constants *a* and *c* of each layer (GaN, Al_x_Ga_1−x_N, AlN) are obtained from Fig. 5 via the equation:


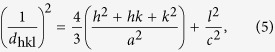


where *d*_hkl_ represents the interplanar spacing of the probed lattice plane (*hkl*)^30^. Employing the Poisson-Vegard’s law^30^ while including the lattice constant bowing parameter^31^, the Al composition (*x*), the free-standing lattice constants (*a*_0_(*x*) and *c*_0_(*x*)), and the in-plane (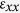) and out-of-plane strain (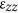) of each layer are calculated via:


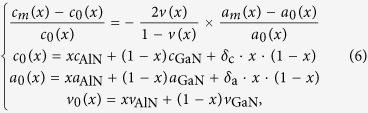



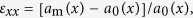



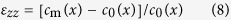


*a*_m_(*x*) and *c*_m_(*x*) are the measured lattice constants obtained from Eq. 5; *a*_AlN_ = 3.112 Å, *c*_AlN_ = 4.982 Å, *a*_GaN_ = 3.186 Å, and *c*_AlN_ = 5.186 Å, representing free-standing lattice constants. The Poisson ratio *v*_AlN_ and *v*_GaN_ are 0.203 and 0.183, respectively^30^. *δ*_c_ and *δ*_a_ represent the deviation (bowing) parameter and were fit to be −0.036 and 0.018 Å, respectively^31^. The in-plane stress (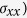 is also calculated according to:


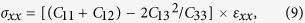


where *C*_ij_ are the elastic constants of GaN (*C*_11_ = 390 GPa, *C*_12_ = 145 GPa, *C*_13_ = 106 GPa, and *C*_33_ = 398 GPa)^32^. The resulting in-plane stress values of GaN layers are tabulated in Table 1. Detailed XRD RSM calculation results, including the lattice constants (*a* and *c*), in-plane strain/stress, and out-of-plane strain of each (Al)GaN layer in all samples, are provided in Supplementary Table SI. The in-plane strain of each (Al)GaN layer of the three samples are also plotted in Supplementary Figure S3. For sample A and B, from the AlN layer toward the Al_x_Ga_1−x_N layers the in-plane strain first decreases from positive (tensile), crossing the zero line (strain-free), and then become negative (compressive). Finally the in-plane strain returns to positive (tensile) due to the growing of the GaN layer. This discovered trend agrees with a previous work^33^ where the in-plane strain in a GaN/Al_x_Ga_1−x_N/AlN system initially decreases along the increasing of the structure thickness and then crosses the zero line; as the thickness of GaN gets thicker the slope gets less steep, suggesting that an in-plane strain with a tensile component launches and becomes more dominant as the GaN thickness gets thicker.

Our XRD RSM results suggest that the GaN layers of all the three samples have tensile stress with sample B having the lowest stress (~0.154 GPa), sample C having the highest one (~1.548 GPa), and sample A having a medium value (~1.059 GPa). This indicates that the step-graded Al_x_Ga_1−x_N layers used in samples A and B reduce the in-plane (tensile) stress of the GaN layer. Moreover, GaN in sample A is shown to have a higher in-plane tensile stress than GaN in sample B. We attribute this to sample A having a thicker GaN layer (a thicker i-GaN layer and an additional n-GaN layer) than sample B. Moreover, it’s been reported that smaller-sized dopants, such as Si, in GaN films will from impurity atmospheres that limit the dislocation movement while growing, which further limits the dislocation reduction and the relief of the GaN tensile stress^34^. At this stage, we fairly believe that the carbon dopant in the n-doped GaN in sample A has an equivalent effect on enlarging the GaN film tensile stress. This indicates the importance of optimal buffer layer configuration in terms of thicknesses and Al-content.

### Investigation of the two dimensional electron gas characteristics via Hall effect measurement

Hall effect measurements under Van der Pauw configuration are carried out at room (300 K) and low (77 K) temperatures. The metal contacts are formed via e-beam evaporation of 200-nm-thick Ti/200- nm-thick Ni metal stack and annealed at 750 °C under N_2_ for 45 seconds. Samples are then diced into 5 mm × 5 mm squares and measured under Van der Pauw configuration.

Carrier concentration (N_s_), sheet resistance (R_s_), and mobility (μ_n_) of samples A, B, and C are tabulated in Table 1. The 2DEG concentration is the highest in sample A, whereas 2DEG mobility is highest in sample B. It is earlier reported that carrier concentration and mobility of a 2DEG system are strongly related^35^. At high 2DEG carrier concentration (> ~ 2 × 10^12^ cm^−3^), the 2DEG mobility has a negative dependence on increasing carrier concentration due to the surface roughness scattering, in agreement with our work. At low temperature (77 K), the 2DEG mobility increases and the sheet resistance decreases in all samples due to the reduced phonon scattering at low (77 K) temperature. Furthermore, 2DEG concentrations in all our samples are higher at 77 K than at 300 K, in line with other works^36^.

## Discussion

Our defectivity measurements via AFM and CL reveal that threading dislocation density reduces with increasing total buffer thickness. We further show step-graded AlGaN buffer layers and thicker GaN buffer layers alone are beneficial in doing so. This is attributed to the interaction of edge- and mixed-type threading dislocations in the forms of fusion (i.e. two threading dislocation lines merging into one) and annihilation (two threading dislocation lines (having opposite Burgers vectors) annihilating one another)^14^. As the (Al)GaN film is further deposited, the possibility of such interactions increases, lowering the threading dislocation density. In particular, the inaccuracy of CL and XRD defectivity measurements are discussed here. As mentioned, CL measurement tend to underestimate the defectivity because these defects act as non-radiative recombination centers that possess capture radius; compared to AFM defectivity the lower defectivity of CL suggests that multiple defects are located within one CL dark capture radius. On the other hand, the estimated XRD defectivity here provides information about the densities of the pure-screw- and pure-edge-type threading dislocations; mixed-type threading dislocation, which typically occupies a large portion of the total defectivity, is not evaluated. In addition, although we conduct asymmetric rocking curve scan on the commonly used 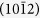 plane to estimate the pure-edge-type threading dislocations, the measured XRD rocking curve FWHM is not solely affected by threading dislocations; other factors such as instrumental broadening, microstrain, and wafer curvature also play roles in changing the FWHM^25^. We suggest to take the AFM defectivity as the total defectivity as it is the most direct and sensitive one, which concludes that sample C should possess a large amount of mixed-type threading dislocations that achieves its largest total defectivity.

X-ray diffraction RSM is used to investigate strain distribution in buffer layers (Fig. 5) providing in-/out-of-plane strain information of GaN, Al_x_Ga_1−x_N, and AlN layers (Table S1). In samples A, B and C, the AlN in-plane tensile strain are found to be 0.47%, 0.51%, and 0.52%, respectively. We observe a gradual reduction of in-/out-of-plane tensile strain in the step-graded 3 × {Al_x_Ga_1−x_N} buffer layers from Si(111) towards the AlGaN/GaN HEMT structure (Table S1 and Figure S3). This leads to a lower tensile GaN in-plane strain in samples A (0.23%) and B (0.03%) than that in sample C (0.34%), showing the importance of step-graded Al_x_Ga_1−x_N buffer layers. Between sample A and B, GaN in-plane tensile strain in sample B is lower than that in sample A leading to a narrower XRD FWHM (Fig. 4). Although Raman spectroscopy reveals a blue shift of GaN 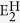 Raman peak in all samples, which corresponds to an in-plane compressive stress (<0.5 GPa), in line with other Raman works^11^. The discrepancy between the stress results from XRD RSM and Raman spectroscopy has been found in previous works^37^,^38^, which is suggested to be attributed to the fact that the stress measured by XRD RSM is an average value throughout the whole structure where the effect brought by local/random lattice distortion and/or any other imperfections are averaged out. On the other hand, the stress investigated by Raman spectroscopy is only from the spot-size area, therefore the value is statistically insufficient and less accurate to represent the strain in the whole GaN layer. In addition, it is reported that the discrepancy will also occur when the material is doped and/or with impurities^37^. Furthermore, stress investigation using Raman Spectroscopy requires a phonon-shift estimation model, which might need to be modified for short period hetero-interfaces such as AlGaN/GaN HEMTs. Thus, we believe XRD RSM to be a more reliable means of probing the stress in thick hetero-interfaced layers than Raman studies.

Hall effect measurements under Van der Pauw configuration are carried out to determine 2DEG concentration, sheet resistance, and 2DEG mobility (Table 1). Sheet resistance is inversely related to the 2DEG mobility, however the relatively large variation of the sheet resistance of sample A and C should be attributed to the surface variation across the samples as the surface morphology is significantly affected by defects. As the defect density and type is varied, it is typical to have a varying surface morphology, and such variation in surface morphology greatly affects the contacts as defects are charged as well as some defects have threading component (during annealing metal is diffused further). The 2DEG concentration is the highest in sample A and the lowest in sample B whereas the 2DEG mobility is the highest in sample B and the lowest in sample A, showing a trade-off between 2DEG concentration and 2DEG mobility^35^. Considering that sample A has the lowest threading dislocations but sample B has the lowest in-plane strain, our work suggests that 2DEG mobility is not only affected by threading dislocations but also by GaN in-plane strain. As 2DEG concentration is affected by the spontaneous (*P*_SP_) and piezoelectric (*P*_PE_) polarizations in the Al_x_Ga_1−x_N barrier layer and the underlying GaN layer (where the piezoelectric polarization is determined by strain^8^), a fixed polarization charge density (C/m^2^) is induced at the AlGaN/GaN hetero-interface given by:


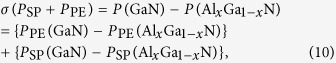


where *P*_SP_ doesn’t change with strain whereas *P*_PE_ follows:


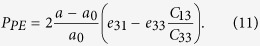


where (*C*_13_, *C*_33_) are elastic constants and (*e*_31_, *e*_33_) are piezoelectric coefficients. For *P*_PE_ (GaN), *a*_0_ = 3.186 Å, *e*_31_ = −0.49 C/m^2^, *e*_33_ = 0.73 C/m^2^, *C*_13_ = 103 GPa, and *C*_33_ = 405 GPa; for *P*_PE_(Al_x_Ga_1−x_N, x = 0.25, 0.23, and 0.22 in sample A, B, and C, respectively), *a*_0_ ≅ 3.165 Å, *e*_31_ ≅ −0.52 C/m^2^, *e*_33_ ≅ 0.92 C/m^2^, *C*_13_ ≅ 104.25 GPa, and *C*_33_ ≅ 397 GPa^8^. Since the Al_x_Ga_1−x_N barrier layer is thin (~17 nm), we fairly assume that it is fully strained with no relaxation, which allows us to further assume that the lattice constant *a* of the Al_x_Ga_1−x_N barrier layer should be the same with that of the GaN layer. From Eqn. 11, we obtain: *P*_PE_(GaN)–*P*_PE_(Al_x_Ga_1−x_N) ≅ 0.057*a*–0.172, suggesting that as lattice constant *a* becomes larger (meaning that both the Al_x_Ga_1−x_N barrier and GaN layers simultaneously stretch further under more tensile strain), {*P*_PE_(GaN)–*P*_PE_(Al_x_Ga_1−x_N)} value and hence the polarization charge density (*σ*) increase. The increase in the polarization charge density results in an increase in 2DEG concentration, in good agreement with our observations. As stated before, the higher 2DEG concentration of sample A and C (due to their larger GaN tensile stress) is responsible for their lower 2DEG mobility because of the severer surface roughness scattering. In summary, we report that GaN layer in-plane tensile strain is the dominant mechanism governing the 2DEG characteristics.

## Conclusion

Scanning electron microscopy, energy-dispersive X-ray spectroscopy, high resolution-cross section transmission electron microscopy, optical microscopy, atomic-force microscopy, cathodoluminescence, Raman spectroscopy, X-ray diffraction (ω/2*θ* scan, symmetric/asymmetric ω scan (rocking curve scan), and reciprocal space mapping), and Hall effect measurements are utilized to investigate AlGaN/GaN HEMT structures on Si(111) with different buffer layer configurations. We show that thicker buffer layers are beneficial for reducing the threading dislocation density, and stacked Al_x_Ga_1−x_N buffer layers are critical for reducing the layer stress. Furthermore, our work suggests that the type and the magnitude of in-plane strain of the GaN layer, not the threading dislocation density, dominate the 2DEG mobility. Overall, we demonstrate the importance of optimal buffer layer configurations for 2DEG characteristics, which might allow high-frequency AlGaN/GaN HEMT designs on Si(111) substrates.

## Methods

### Sample preparation and cleaning

The AlGaN/GaN heterostructures used for this investigation are cut from three different 200-mm-diameter wafers (A, B, and C) with Si(111) substrate. The epitaxy layer configurations are shown in Fig. 1. Samples (8 pieces of sample A from the wafer A, 18 pieces of sample B from the wafer B, and 6 pieces of sample C from the wafer C) populate on the wafer approximately ~15 cm away from the center. Sample cleaning is carried out as follows: Samples are soaked in (1) hot trichloroethylene (TCE), (2) hot acetone, (3) hot methanol followed by (4) DI rinse and (5) N_2_ blow dry. For steps (1–3), an ultrasonic bath cleaning is added. All steps are carried out for ~10 minutes.

### Metallization, annealing, and dicing of AlGaN/GaN HEMT structures for Hall measurements

After sample cleaning, 200-nm titanium (Ti) and then 200-nm nickel (Ni) metal contacts were deposited through a square-shaped patterned hard mask on the Hall mobility measurement samples. The metal deposition rates were kept between 1.5 and 2.0 Å/s. Rapid thermal annealing was carried out at 750 °C for 45 s under N_2_ for ohmic contact formation. Then, photoresist (PR) (AZ 5214) was spun on the samples then soft-baked at 95 °C for ~10 min in order to prevent scratching or damaging the sample surface during dicing. Finally, nickel-bond dicing blade (0.051 mm thick, 3–6 micron diamond grain size) mounted on K&S 708 dicing saw is used for dicing samples into small square dies with metal contacts in the four corners. Samples were re-cleaned before measurements. Hall measurement were performed three times for each piece of sample. Finally we calculate the overall averages and standard deviations of the measured data from these three kinds of samples.

## Additional Information

**How to cite this article**: Lee, H.-P. *et al*. Investigation of AlGaN/GaN high electron mobility transistor structures on 200-mm silicon (111) substrates employing different buffer layer configurations. *Sci. Rep.* **6**, 37588; doi: 10.1038/srep37588 (2016).

**Publisher’s note**: Springer Nature remains neutral with regard to jurisdictional claims in published maps and institutional affiliations.

## Supplementary Material

Supplementary Information

## Figures and Tables

**Table 1:** Structural (surface roughness, defectivity, stress) and electrical (contact resistance, 2DEG concentration, 2DEG mobility) properties of samples A, B, and C are tabulated.

Sample (Epi thickness)	AFM surface roughness (Å)	Defectivity (10^9^ cm^−2^)				GaN stress (GPa)		Electrical property					
		AFM	CL	XRD		XRD	Raman	*R*_s_ (Ω/◽)		2DEG *N*_s_ (10^13^ cm^−2^)		2DEG *μ*_n_ (cm^2^/V-s)	
				*D*_screw_	*D*_edge_	Tensile	Compressive	RT	77 K	RT	77 K	RT	77 K
A (6.3 μm)	5.5 ± 2.8	1.8 ± 0.3	1.0 ± 0.3	0.40	1.02	1.059	0.256	431.8 ± 109.8	110.8 ± 28.1	1.20 ± 0.01	1.30 ± 0.10	1295 ± 247	4867 ± 996
B (2.0 μm)	5.2 ± 1.4	2.2 ± 0.8	2.0 ± 0.3	0.58	1.47	0.154	0.488	385.8 ± 28.1	66.5 ± 5.0	0.89 ± 0.01	1.00 ± 0.01	1802 ± 102	9175 ± 698
C (1.1 μm)	5.1 ± 1.3	2.6 ± 0.8	1.8 ± 0.3	0.39	1.02	1.548	0.047	445.5 ± 75.3	96.0 ± 23.0	0.92 ± 0.05	1.00 ± 0.10	1593 ± 268	6930 ± 1541
