# Supplementary material for: Investigation of AlGaN/GaN high electron mobility transistor structures on 200-mm silicon (111) substrates employing different buffer layer configurations
Source: Sci Rep. 2016 Nov 21;6:37588. doi: 10.1038/srep37588 (PMC5116587; doi:10.1038/srep37588)
Supplement: Supplementary Information [file srep37588-s1.pdf]

Supplementary Online Material for: "Investigation of AlGaN/GaN high electron mobility transistor structures on 200-mm silicon (111) substrates employing different buffer layer configurations"

H.-P. Lee[1, 2]**, J. Perozek**[1, 2]**, L. D. Rosario**[1, 2] **& C. Bayram*** [1, 2]

[1]Department of Electrical and Computer Engineering, University of Illinois at Urbana-Champaign, Urbana, Illinois 61801, USA

[2]Micro and Nanotechnology Laboratory, University of Illinois at Urbana-Champaign, Urbana, Illinois 61801, USA

*cbayram@illinois.edu

This file includes:

- Figure S1
- Figure S2
- Table SI
- Figure S3

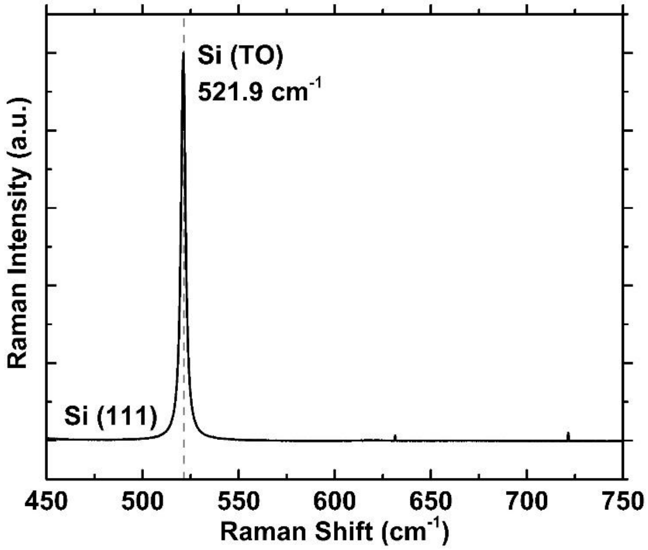

Figure S1. Si (111) Raman spectrum measured by Horiba Raman Confocal Imaging Microscope using 633-nm laser line with a grating of 1800 lines/mm, yielding a spectrum resolution of 1.19 cm^{-1}. A 521.9 cm^{-1} Si (111) Raman shift was observed.

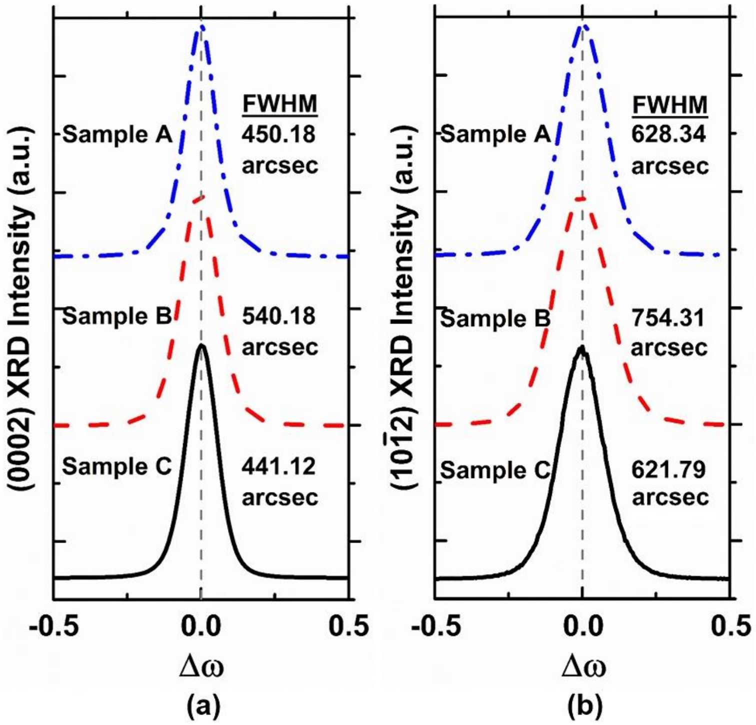

Figure S2. (a) Symmetric and (b) asymmetric ω (rocking curve) scans of (0002) and $(10\bar{1}2)$ lattice planes are shown. Symmetric ω scan on (0002) plane is used for investigating the screw-type threading dislocation density and asymmetric ω scan on $(10\bar{1}2)$ plane is used for edge-type threading dislocation density investigation (Table 1).

Sample (Epi thickness)	Layers	Measured lattice constant (*a*/*c*) (Å)	Free-standing lattice constant (a_0/c_0) (Å)	In-plane strain/stress (%)/(GPa)	Out-of-plane strain (%)
A (6.315 μm)	GaN	3.1936/5.1827	3.1860/5.1860	+0.2331/+1.0586	-0.0629
	$Al_{0.33}Ga_{0.67}N$	3.1558/5.1275	3.1574/5.1262	-0.0540/-0.2364	+0.0253
	$Al_{0.60}Ga_{0.40}N$	3.1371/5.0730	3.1375/5.0728	-0.0067/-0.0283	+0.0032
	$Al_{0.82}Ga_{0.18}N$	3.1295/5.0176	3.1224/5.0232	+0.2246/+0.9185	-0.1119
	AlN	3.1266/4.9678	3.1120/4.9820	+0.4699/+1.8659	-0.2849
B (2.007 μm)	GaN	3.1873/5.1855	3.1860/5.1860	+0.0340/+0.1544	-0.0088
	$Al_{0.30}Ga_{0.70}N$	3.1567/5.1295	3.1583/5.1283	-0.0487/-0.2140	+0.0228
	$Al_{0.58}Ga_{0.42}N$	3.1365/5.0726	3.1372/5.0720	-0.0250/-0.1059	+0.0121
	$Al_{0.82}Ga_{0.18}N$	3.1290/5.0161	3.1220/5.0217	+0.2254/+0.9218	-0.1124
	AlN	3.1279/4.9674	3.1120/4.9820	+0.5134/+2.0387	-0.2933
C (1.125 μm)	GaN	3.1968/5.1815	3.1860/5.1860	+0.3408/+1.5477	-0.0872
	AlN	3.1283/4.9670	3.1120/4.9820	+0.5181/+2.0573	-0.3004

Table S1. XRD reciprocal space mapping calculations are tabulated. The free-standing lattice constants (a_0/c_0) and elastic constants of $Al_xGa_{1-x}N$ are calculated using Poisson-Vegard's law with bowing parameter correction.[1,2]

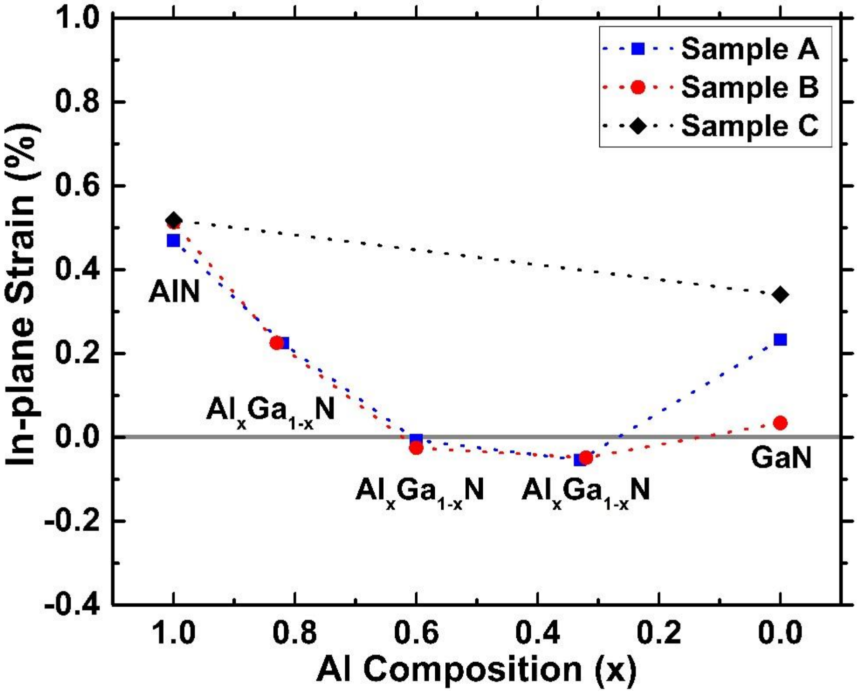

Figure S3. In-plane strain of GaN, $Al_xGa_{1-x}N$, and AlN obtained from XRD reciprocal space mapping are demonstrated. For sample A and B, from the AlN layer to the $Al_xGa_{1-x}N$ layer the in-plane strain first decreases from positive (tensile), crossing the zero line (strain-free), and then become negative (compressive). Finally the in-plane strain returns to positive (tensile) due to the growing of the GaN layer. Sample A has a thicker GaN layer than sample B, which results in the higher in-plane tensile strain of the GaN layer. Without any $Al_xGa_{1-x}N$ buffer layer, the GaN in-plane tensile strain of sample C is investigated to be the largest one.

References

1. Kadir, A., Huang, C.-C., Lee, K. E.-K., Fitzgerald, E. A. & Chua, S.-J. Determination of alloy composition and strain in multiple AlGaN buffer layers in GaN/Si system. *Appl. Phys. Lett.* **105**, 232113 (2014).
2. Liou, B.-T., Yen, S.-H., & Kuo, Y.-K., Vegard's law deviation in band gaps and bowing parameters of the wurtzite III-nitride ternary alloys. Proc. SPIE 5628, Semiconductor Lasers and Applications II, (20 January 2005).
